# From loss to disorder: The influence of maladaptive coping on prolonged grief

**DOI:** 10.1016/j.psychres.2024.116060

**Published:** 2024-09

**Authors:** Kirsten V. Smith, Jennifer Wild, Anke Ehlers

**Affiliations:** a Department of Experimental Psychology, University of Oxford, UK; b Oxford Health NHS Foundation Trust, Oxford, UK; c The Loss Foundation, [Registered Charity 1147362], London, UK; d Phoenix Australia Centre for Posttraumatic Mental Health, Department of Psychiatry, University of Melbourne, Melbourne, VIC, Australia

**Keywords:** Prolonged grief disorder, Bereavement, Posttraumatic stress disorder, Coping strategies, Structural equation modelling, Cross-lagged panel models, Proximity seeking, Rumination, Avoidance, Continuing bonds, Psychometrics, Confirmatory factor analyses, Exploratory factor analyses

## Abstract

Research indicates that post-bereavement coping strategies can be adaptive or maladaptive. Understanding which strategies lead to poorer outcomes is an important clinical and theoretical question with the potential to guide intervention.

The Oxford Grief – Coping Strategies scale was developed from interviews with bereaved people with and without prolonged grief disorder (PGD) to assess the frequency of maladaptive cognitive and behavioural strategies after bereavement. Factorial and psychometric validity were assessed using exploratory and confirmatory factor analysis (*N* = 676). A three-wave cross-lagged panel model (*N* = 275) was used to assess the predictive validity of the tool in explaining symptoms of PGD.

Results supported a four-factor solution (Avoidance, Proximity Seeking, Loss Rumination, Injustice Rumination) with good psychometric properties. The OG-CS predicted prospective symptoms of PGD in the short-term (6–12 months) and long term (12–18 months), controlling for baseline symptoms and autocorrelations. Subscale analyses demonstrated that the use of coping strategies predicted ICD-11 PGD in both the short-term and the long-term. However, avoidance was not predictive of outcomes early in the grieving process. At 6–12 months, avoidance predicted PGD at 12–18 months.

## Highlights

• The type of coping strategy utilised in the early months of loss has a significant impact on later symptoms of prolonged grief disorder (PGD).
• Use of avoidant strategies, proximity seeking strategies, and rumination measured at 6–12 months all significantly predict prospective symptoms of prolonged grief disorder at 12–18 months.
• Use of avoidant coping strategies in the early months after loss (0–6 months) did not predict elevated PGD 6 months later.

## 1 Introduction

Epidemiological research identifies loss of a loved one as one of the most common lifetime stressful experiences (Kessler et al., 2017). Following such an event, many individuals will adapt. However, approximately up to 10%, will go onto develop prolonged grief disorder (PGD) a condition associated with significant individual and societal cost, making the understanding of the onset and maintenance of PGD a critical public health issue (Lundorff et al., 2017).

Cognitive behavioural models of PGD suggest that specific coping strategies, such as avoidance or rumination, play a key role in maintaining symptoms (see Eisma and Stroebe, 2021 for a review). These strategies, often understandably employed post-bereavement, may hinder the processing of the loss and indeed research has established a link between post-loss coping strategies and adverse outcomes after loss (Boelen and Eisma, 2015; Eisma et al., 2015; Morina, 2011; Nam, 2016; Shear et al., 2007; Smith and Ehlers, 2020, 2021b; Stroebe et al., 2007). Theoretical models propose that such coping strategies are counterproductive and impede the integration of the memory of the loss into autobiographical memory, reinforcing negative appraisals formed after loss (Boelen, van den Hout, et al., 2006; Duffy and Wild, 2023; Ehlers and Clark, 2000). However, to our knowledge only two studies have directly explored the relationship between these cognitive behavioral factors and PGD symptoms longitudinally after a bereavement. Smith and Ehlers (2020) identified four trajectories of grief, three of which demonstrated a link between negative appraisals and memory characteristics in predicting unhelpful coping strategies. This pattern of results was not found in a fast adaptation group, who made less use of unhelpful coping strategies, perhaps explaining their relatively quick reduction in grief symptoms. Another study (Smith and Ehlers, 2021a) demonstrated that early memory characteristics and negative appraisals predict later unhelpful coping strategies, which fully mediated their impact on PGD symptoms at 12–18 months post-loss. These findings underscore the pivotal role of post-loss coping strategies in the development and maintenance of bereavement-related mental health problems. However, an aspect that was not controlled for in Smith and Ehlers (2021a), due to model complexity, was the influence of autocorrelations, leaving open the question whether unhelpful coping utilised in the early months of loss can predict later symptoms of PGD after controlling for baseline symptoms.

The coping strategies measured in these studies (Smith and Ehlers, 2020, 2021a) were derived through a review of the literature (Boelen et al., 2003; Eisma et al., 2014; Field and Filanosky, 2009; Field et al., 2003; Mancini et al., 2009) and from interviews with bereaved people with and without PGD (Smith, 2018). Unlike existing scales that have tended to measure one category of coping (Baker et al., 2016; Boelen and van den Bout, 2010; Eisma and Nguyen, 2023; Eisma et al., 2014) the Oxford Grief – Coping Strategies scale (OG-CS), measures a broad range of strategies (e.g. avoidance behaviours, thought suppression, proximity seeking behaviours, and rumination about a range of topics). It was designed to be a concise, yet comprehensive, tool for use in clinical settings to plan and guide treatment. The scale's use in previous studies was to investigate its role in combination with other proposed cognitive behavioural mechanisms of PGD. In this paper we present its psychometric validity and take a closer look at the types of coping strategies relevant to severe and enduring grief.

Previous research, has demonstrated that each type of coping strategy measured in the OG-CS is linked to the development and/or maintenance of PGD. Boelen and Eisma (2015) discovered that in the first year of loss, using anxious avoidant strategies (i.e., avoiding confronting the loss's reality) predicted PGD after one year. Similarly, in a study of the bereaved family members of those killed in the Utøya terrorist attack, researchers found that avoidance of reminders of the loss predicted a high and chronic grief trajectory up to 40 months later (Kristensen et al., 2020). Using the Utrecht Grief Rumination Scale (UGRS) to identify adaptive and maladaptive rumination, Eisma et al. (2015) demonstrated that ruminating on the perceived injustice of the loss predicted additional variance in PGD symptoms after controlling for baseline symptoms. Subsequent research linked counterfactual thinking (i.e., dwelling on what might have prevented the death) to concurrent and longitudinal PGD and depression symptoms (Eisma et al., 2021). Rumination as a cognitive coping strategy has also been proposed as a key mechanism of change in treatment studies (Glickman et al., 2017).

Proximity seeking (i.e. aiming to restore a connection with the deceased) reflects a process also known as continuing bonds (Field et al., 2003). Longitudinal studies historically linked continuing bonds to increased grief symptoms (Boelen, Stroebe, et al., 2006; Field et al., 2003). Recent research by Eisma and Nguyen (2023) found concurrent associations of proximity seeking with prolonged grief (PG), but so far no longitudinal investigations have investigated the potential role of proximity seeking in the onset and maintenance of PGD.

Since unhelpful coping strategies are central to theoretical models of PGD it is essential to understand how and when these strategies influence the development and persistence of the disorder. Furthermore, it is especially important to identify specific maladaptive strategies given that avoidance, proximity seeking and rumination are commonplace in the acute phase of grief and can be considered part of a normative grieving process (Eisma et al., 2015; Shear et al., 2007; Stroebe and Schut, 1999). Careful characterisation of the mechanisms of PGD have the potential to inform interventions aimed at alleviating distress after loss. Previous research has shown that PGD symptoms reduce when participants are guided through interventions to adopt more adaptive coping strategies. Using Prolonged Grief Treatment, formerly Complicated Grief Treatment, Shear and colleagues found that reductions in avoidant coping mediated reductions in grief symptoms and impairment (Lechner-Meichsner et al., 2022). A further randomised controlled trial using metacognitive therapy for grief (MCGT) directly targeted ruminative coping and found significant treatment effects on PGD symptoms and reductions in rumination (Wenn et al., 2019). These findings highlight the importance of thorough assessment of maladaptive coping as a means for guiding therapeutic work.

The present study utilises two samples of bereaved adults recruited from the community to test the factorial and psychometric properties of the Oxford Grief Coping Strategies Scale (OG-CS) using exploratory and confirmatory factor analyses. A separate three wave longitudinal sample assessed in the first 6 months of loss and then followed at 6 and 12 months later was used to test the predictive utility of the OG-CS in explaining symptoms of PGD over time. Cross-lagged panel analyses controlling for the effect of prior symptoms levels and concurrent associations was employed to elucidate longitudinal effects.

## 2 Methods

### 2.1 Participants

Psychometric validation utilized two separate samples (cross-sectional, *N* = 676; and test-retest, *N* = 50) of adults bereaved at least 6 months earlier. Longitudinal investigations involved a third separate sample (*N* = 275) assessed within the first 6 months of their bereavement (baseline 0–6 months) and followed up in the short term (6–12 months) and long-term (12–16 months). All participants (recruited through bereavement charity mailing lists, social media advertisments, and keyword targeted advertising on the Google content network) were compensated for their time (£10 per survey). Participants were included if the deceased was a close loved one. Table 1 displays each sample's demographic data and loss characteristics. The test-retest sample completed measures 1 week apart.

**Table 1:** Sample demographics and loss characteristics

Variable	Sample		
	Cross-sectional	Test-retest	Longitudinal
	(*N* = 676)	(*N* = 50)	(*N* = 275)
Age M (SD)	49.22 (12.52)	51.46 (14.54)	46.43 (13.24)
Women (%)	81.5	84.0	78.5
Months since loss M (SD)	56.91 (79.79)	23.74 (48.44)	2.94 (2.01)
Violent loss (%)	19.5	26.0	9.1
Who died? (%)			
Partner	36.1	28.0	30.2
Child	21.0	22.0	8.7
Sibling	6.5	0.0	5.8
Parent	28.3	42.0	38.2
Another close relative or non-relative	8.2	8.0	17.1

Note: Violent loss defined as resulting from human (in)action (i.e. suicide, homicide, accident, unintentional overdose, medical negligence) versus illness.

### 2.2 Procedure

Questionnaire data were collected online (Qualtrics, Provo, UT) after providing informed consent in accordance with ethical approval given by the University of Oxford Medical Sciences Inter-Divisional Research Ethics Committee (MS-IDREC—C1–2015–230; MS-IDREC—C1- 2015–231). Questionnaires tailored items to the user; participants selected a preferred name for the deceased, integrated into relevant items (e.g., ‘I bring images of Maggie to mind’). In line with ethical guidelines, participants received a post-questionnaire email offering support and a chance to discuss any distressing aspects (Smith et al., 2018).

### 2.3 Measures

The OG-CS was designed to be a comprehensive assessment of unhelpful coping strategies after loss. Individual items and content domains were developed in collaboration with cognitive therapists experienced in the treatment of chronic grief reactions and through a review of the literature (Boelen et al., 2003; Eisma et al., 2014; Field and Filanosky, 2009; Field et al., 2003; Mancini et al., 2009). Some items from the English version of the UGRS measuring injustice rumination and loss rumination were adapted for use in the scale given their strong predictive utility in PGD (Eisma et al., 2015). Item selection involved evaluating mean scores, correlations with PGD symptoms, and removal of low-scoring items. Specialist therapists and bereaved representatives reviewed final items for content and face validity.

#### 2.3.1 Cognitive measures

*The Oxford Grief Coping Strategies Scale (OG-CS).* The OG-CS is a 23-item questionnaire assessing the frequency of loss-related coping strategies utilised in the past month on a 5-point scale (1 = *never*, 5 = *always*). The four content domains include avoidance (6 items, e.g. “I avoid places we went together”), proximity seeking (7 items, e.g. “I am still carrying out a routine as a way of caring for them”), loss rumination (7 items, e.g. “I dwell on moments that could have changed the outcome”), and injustice rumination (3 items, “I think over and over about how it could be that this happened”). Internal consistency was acceptable in the cross-sectional sample (*N* = 676, ω = 0.79) and good in the longitudinal sample (*N* = 275, ω=0.87)

#### 2.3.2 Symptom measures

##### 2.3.2.1 Prolonged grief disorder inventory

The PG-13; Prigerson and Maciejewski (2008) assessed symptoms of separation distress, cognitive, emotional and behavioural problems and their intensity and duration following a bereavement. We used an extended version that covered the 10 symptoms of DSM-5-TR diagnostic criteria (see Smith et al., 2022 for a fuller description). A probable PGD diagnosis required reporting at least one item of separation distress and a minimum of three daily cognitive, emotional, or behavioral disturbances from a possible eight symptoms, significantly impairing functioning in one or more life areas (social, occupational, domestic responsibilities) (Prigerson et al., 2009). For the ICD-11 criteria symptoms (Killikelly and Maercker, 2018) we included item 14 from the PCL-5 “trouble experiencing positive feelings” rescaled to match the PG-13 items.

##### 2.3.2.2 Posttraumatic stress disorder checklist for DSM-5 (PCL-5) (Weathers et al., 2013)

Consisting of four scales corresponding to DSM-5 PTSD symptom clusters, the PCL-5 includes items to assess re-experiencing, avoidance, negative alterations, and hyper-arousal symptoms. Twenty self-report items are scored 0–4, with a recommended cut-off score of 33 for a probable PTSD diagnosis. Participants referred to the death of their significant other in completing the PCL.

##### 2.3.2.3 *Patient health questionnaire (PHQ-9)* (Kroencke et al., 2001)

The PHQ-9 is a nine item self-report measure of general depression and distress based on the criteria for major depressive disorder (DSM IV-TR, American Psychiatric Association, 2000). Participants rate the frequency of depressive symptoms over the past two weeks on a scale of 0 (not at all) to 3 (nearly every day) with total scores ranging from 0 to 27.

### 2.4 Data analysis

#### 2.4.1 Statistical analyses

Two-stage factor analyses were performed, utilizing a 50% random split for exploratory factor analyses (EFA) to build the measurement model and the remaining half for cross-validation via confirmatory factor analyses (Osborne and Fitzpatrick, 2012). Geomin oblique rotation was employed due to expected correlations among scale factors (Muthén and Muthén, 2007). The weighted least squares mean and variance adjusted (WLSMV) estimation method was chosen, treating the 5-point questionnaire data as ordered categorical (Kaplan, 2008; Muthén and Muthén, 2007).

Conceptual interpretability guided model adequacy assessment. Criteria included a χ2:df ratio smaller than 3:1, a comparative fit index (CFI) of 0.90 or higher (acceptable) and 0.95 or higher (good), and a root mean square error of approximation (RMSEA) of 0.08 or lower (acceptable) and 0.06 (good) (Hu and Bentler, 1999). Parallel analyses generated scree plot results, eigenvalues greater than 1, and loadings > 0.35 determined factor membership. Cross-loading items were placed where most conceptually sensible. Modification indices over 30 were considered if conceptually interpretable (Brown, 2014). Lastly, a higher-order factor score was modelled to support future use of a sum score (Brown, 2014).

#### 2.4.2 Psychometric validation

McDonald's Omega (ω = (Σ|λi |)² / ([Σ|λi |]² + Σδii)) gauged composite reliability for WLSMV on the total scale and individual factors from EFA (Gadermann et al., 2012).

Criterion and convergent validity were assessed through correlations with psychopathology measures (PGD, PTSD, depression). Factorial convergent validity used the average variance extracted (AVE), (Fornell and Larcker, 1981), with a threshold of 0.50 or higher (Hair et al., 2011). Factorial discriminant validity was confirmed if AVE exceeded the highest squared inter-construct correlation (Henseler et al., 2015). A test re-test reliability correlation of >0.70 over 7 days indicated scale stability.

#### 2.4.3 Structural equation modelling

##### 2.4.3.1 OG-CS

**Fig. 1:**
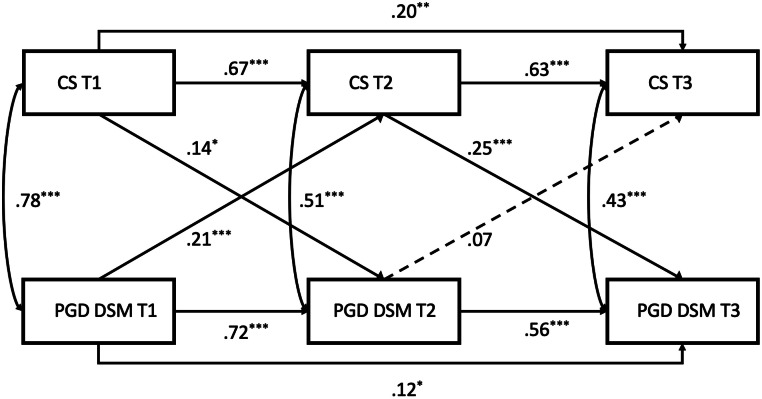
PGD DSM, χ^2^ =0.87, *df* =2, *p* > .05, RMSEA=0.00 (0.00–0.09), CFI = 1.00 TLI = 1.00.

**Fig. 2:**
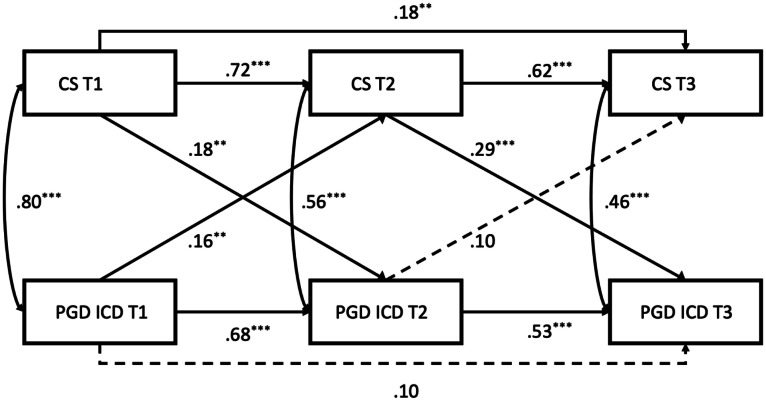
PGD ICD-11 χ^2^ = 0.38, *df* = 2, *p* > 0.05, RMSEA = 0.00 (0.00–0.07), CFI = 1.00, TLI=1.00.

Second-order autoregressive cross-lagged panel models in Mplus Version 8 (Muthén and Muthén, 2007) were used to assess the influence of coping strategies on PGD symptoms. Models utilised ICD-11 and DSM-5TR criteria for PGD and assessed coping strategies' impact at baseline (0–6 months), short-term follow-up (6–12 months), and long-term follow-up (12–18 months). Autoregressive paths and correlated errors considered influences over time. Model constraints evaluated coping strategies' impact on symptoms over time (see Figs. 1 and 2). Model constraints evaluated coping strategies' impact on symptoms over time.

##### 2.4.3.2 Subscales of the OG-CS

Cross-lagged analyses, using the sum scores of each subscale (Avoidance, Proximity Seeking, Loss Rumination, Injustice Rumination), assessed their influence on PGD symptoms over time. Covariance coverage met the convergence threshold (0.10), ranging from 0.67 to 0.99 for each variable pair. Full information maximum likelihood (FIML) handled minimal missing data at each time point (Baseline=99%, short-term FU = 75%, long-term FU= 78%) allowing all 275 observations to be used. Adequate fit criteria were χ2 *p* > 0.05, CFI > 0.90, TLI > 0.90, and RMSEA < 0.01 (Hu and Bentler, 1999; MacCallum et al., 1996; Wickrama et al., 2016).

## 3 Results

### 3.1 Exploratory factor analyses – coping strategies

All 23 coping strategy items underwent EFA using WLSMV estimation, indicating a four-factor structure with eigenvalues greater than 1. The scree plot supported a three or four-factor solution. The fit statistics for the three-factor solution had a good CFI at 0.97 and adequate RMSEA = 0.068 and χ^2^ = 463.06 on df = 187, χ^2^:df = 2.48. Both four (CFI = 0.98, RMSEA = 0.056, χ^2^ = 336.15 on df = 167, χ^2^:df = 2.01) and five-factor solutions (CFI = 0.99, RMSEA = 0.041, χ^2^ = 230.49 on df = 148, χ^2^:df = 1.56) indicated good model fit. Considering eigenvalues, fit statistics, and weak loading items, a four-factor solution was deemed best. Factors were labeled 'avoidance,' 'proximity seeking,' 'loss rumination,' and 'injustice rumination.' Two items cross-loaded above 0.35 on two factors but were placed based on the strongest loading. The coping strategies items and respective standardised factor loadings are presented in Table 2.

**Table 2:** Factor analyses of the coping strategies scale.

		Factors							
		1		2		3		4	
Coping strategies items		EFA	CFA	EFA	CFA	EFA	CFA	EFA	CFA
1	I avoid watching television programmes that remind me of [-] or death in general.	.61	.62						
2	I avoid places we went together.	.84	.58						
3	I avoid eating foods and meals that we shared or [-] liked.	.79	.65						
4	I avoid making any changes to my life since [-] ’s death.	.44	.71						
5	I make an effort to hold back my feelings.	.39	.51						
6	In the company of others I try hard to stop myself from breaking down.	.39	.67						
7	I feel compelled to surround myself with things that they liked.			.81	.69				
8	I bring images of [-] to mind.			.72	.59				
9	I am still carrying out a routine as a way of caring for them.			.55	.66				
10	I neglect other things because I spend a lot of time doing things for [-] (e.g. creating memorials, fundraising).			.42	.76				
11	I feel compelled to touch things that [-] touched (e.g. belongings, chairs, beds).			.79	.73				
12	I spend a lot of time thinking about joining [-] (in the afterlife).			.40	.78				
13	I dwell on the things we won't get to do together.			.45	.84				
14	I can't stop thinking about how afraid [-] was.					.58	.75		
15	I think over and over about how others failed to ease their suffering.^a^					.76	.77		
16	I think over and over about what I could have done to prevent [-]'s death/ease their suffering.^a^					.95	.86		
17	I go over and over how our last moments could have been more fulfilling.					.47	.70		
18	I dwell on moments that could have changed the outcome.					.81	.81		
19	I can't stop thinking about how much [-] suffered.					.68	.83		
20	I worry that [-] has not found peace (in the afterlife).					.59	.74		
21	I ask myself why I deserved this loss.^a^							.69	.71
22	I think about the unfairness of the loss.^a^							.78	.87
23	I think over and over about how it could be that this happened.							.49	.87
									
Correlations matrix of OG-CS factors									
	Factor 1	–	–						
	Factor 2	.49	.73	–	–				
	Factor 3	.47	.63	.51	.65	–	–		
	Factor 4	.44	.57	.52	.65	.64	.72	–	–
				
Higher order – Coping strategies subscale loadings		.80		.82		.82		.91	

Note: EFA (*N* = 348) CFA (*N* = 328). Factors labelled as follows: 1. Avoidance, 2. Proximity seeking, 3. Loss rumination, 4. Injustice rumination. All factor loadings significant to *p* < .05.

a Adapted Utrecht Grief Rumination Scale items (Eisma et al., 2014).

### 3.2 Confirmatory factor analyses – coping strategies

The CFA, assessing the chosen four-factor solution in a separate sample (*N* = 328), indicated good model fit (CFI = 0.96, RMSEA = 0.061, χ2 = 474.68, df = 224, χ2:df = 2.12). A significant chi-square difference test favoured the four-factor model over three factors (χ2 = 683.03, df = 6, *p* < .001). A higher-order factor solution, combining subscale factors into a single 'coping strategies' factor, showed good fit (CFI = 0.95, RMSEA = 0.06, χ2 = 502.19, df = 226, χ2:df = 2.22). All four-factor loadings were statistically significant (*p* < .001), supporting the use of overall and subscale factor scores. Table 2 summarises the standardised factor loadings for the four-factor and higher order factor solutions for the EFA and CFA, and the inter factor correlation matrix. Fitting the CFA model required no constraints on residual correlations.

### 3.3 Psychometric validation

Composite reliability and internal consistency for the total OG-CS and its subscales were good or excellent, except avoidance, which was acceptable. Test-retest reliability for the total scale was good and subscale reliability was either acceptable or good. Validity and reliability metrics are reported in Table 3 Correlations between the total score of the OG-CS, its subscales, and symptom measures of PGD, PTSD, and depression were all moderate or strong and significant, confirming criterion validity. The avoidance subscale did not meet the requirements for factorial convergent validity (AVE > 0.5) and discriminant validity (AVE > max *r*^2^). However, convergent and discriminant validity was confirmed for all other subscales.

**Table 3:** Psychometric validity of total coping strategies scale and latent factors.

			Factors			
Reliability/ Validity	Measure	Total scale	1	2	3	4
Composite	McDonald's Omega	.96	0.79	.88	.92	.85
Criterion	PGD *r*	.72⁎⁎⁎	.61⁎⁎⁎	.61⁎⁎⁎	.57⁎⁎⁎	.61⁎⁎⁎
	PTSD *r*	.74⁎⁎⁎	.67⁎⁎⁎	.56⁎⁎⁎	.64⁎⁎⁎	.58⁎⁎⁎
	Depression *r*	.61⁎⁎⁎	.59⁎⁎⁎	.50⁎⁎⁎	.48⁎⁎⁎	.48⁎⁎⁎
Test-retest	*r*	.86⁎⁎⁎	.79⁎⁎⁎	.76⁎⁎⁎	.89⁎⁎⁎	.82⁎⁎⁎
Convergent	AVE		.39	.53	.61	.67
Discriminate	Largest Inter-construct *r*^2^		.53	.52	.52	.52

Note: Factors labelled as follows: 1. Avoidance, 2. Proximity seeking, 3. Loss rumination, 4. Injustice rumination. *r =* correlation. Test-retest reliability confirmed if *r* > 0.70. Convergent validity of factors confirmed if AVE > 0.5. AVE = Average variance extracted. Factorial discriminant validity confirmed if AVE > Largest inter-construct *r*^2^.

⁎⁎⁎ *p* < .001.

### 3.4 Cross lagged models

All cross-lagged models demonstrated excellent fit (see Figures for details). Figs. 1 and 2 show parameter estimates for PGD ICD11 and DSM-5TR respectively. Unhelpful coping strategies significantly predicted symptoms of PGD (in both ICD11 and DSM-5TR conceptualisations) 6 and 12 months later after controlling for symptoms at the preceding time point. Although the influence of prior coping strategies on PGD appears to increase in magnitude over time this difference was not significant (PGD ICD-11, β = 0.08, SE= 0.05, *p* > 0.05; PGD DSM, β = 0.07, SE= 0.04, *p* > 0.05.

Looking at the reverse direction of prediction over time, baseline symptoms of PGD predicted coping strategies only in the short term (6–12 months).

### 3.5 Cross lagged models of the subscales of Oxford grief coping strategies scale

The structural models of the cross-lagged analyses for each OG-CS subscale are presented in the supplementary analyses. All models had an excellent fit to the data. The avoidance subscale did not predict ICD-11 or DSM-5-TR PGD symptoms in the short-term (6–12 months), only in the long-term (12–18 months) (PGD-ICD11, β = 0.12, S.E. = 0.05, *p* = .021; PGD-DSM5, β = 0.12, S.E. = 0.05, *p* = .019). The effect of PGD symptoms on avoidance strategies was significant in both the short-term and the long-term (Short-term PGD-ICD11, β = 0.26, S.E. = 0.06, *p* < .001; Long-term PGD-ICD11, β = 0.15, S.E. = 0.06, *p* = .014; Short-term PGD-DSM5, β = 0.34, S.E. = 0.06, *p* < .001; Long-term PGD-DSM5, β = 0.15, S.E. = 0.06, *p* = .019).

Proximity seeking strategies predicted higher PGD symptoms both in the short term (PGD-ICD11, β = 0.11, S.E. = 0.05, *p* = .021, PGD-DSM5, β = 0.12, S.E. = 0.05, *p* < .001) and the long term (PGD-ICD11, β = 0.22, S.E. = 0.05, *p* < .001, PGD-DSM5, β = 0.21, S.E. = 0.05, *p* < .001). In the opposite direction, PGD symptoms also predicted higher proximity seeking strategies both in the short and long term (Short-term PGD-ICD11, β = 0.16, S.E. = 0.06, *p* = .006; Long-term PGD-ICD11, β = 0.16, S.E. = 0.06, *p* = .004; Short-term PGD-DSM5, β = 0.20, S.E. = 0.06, *p* < .001; Long-term PGD-DSM5, β = 0.16, S.E. = 0.06, *p* = .007)

Loss rumination significantly predicted PGD symptoms in the short term for the ICD-11 criteria (β = 0.10, S.E. = 0.05, *p* = .042) and at trend level for DSM-5-TR (β = 0.08, S.E. = 0.04, *p* = .068) and for both conceptualisations in the long term (PGD-ICD11, β = 0.15, S.E. = 0.05, *p* = .002; PGD-DSM5, β = 0.13, S.E. = 0.05, *p* = .003). PGD symptoms only predicted higher loss rumination in the short term (PGD-ICD11, β = 0.19, S.E. = 0.05, *p* = .001; PGD-DSM5, β = 0.20, S.E. = 0.05, *p* < .001) but not the long-term.

The same pattern was observed for injustice rumination in that it predicted PGD symptoms in the short term for the ICD-11 criteria (β = 0.14, S.E. = 0.05, *p* = .006) and at trend level for DSM-5-TR (β = 0.09, S.E. = 0.05, *p* = .062) and for both conceptualisations in the long term (PGD-ICD11, β = 0.17, S.E. = 0.05, *p* = .001; PGD-DSM5, β = 0.15, S.E. = 0.05, *p* = .002). In the opposite direction, PGD symptoms only predicted higher injustice rumination in the short-term (PGD-ICD11, β = 0.19, S.E. = 0.06, *p* = .001; PGD-DSM5, β = 0.18, S.E. = 0.05, *p* = .001) but not the long-term.

## 4 Discussion

The OG-CS was found to have four empirically supported factors (i.e. Avoidance, Proximity Seeking, Loss rumination, Injustice rumination). Each factor demonstrated good or excellent internal consistency, test-retest reliability, and discriminant validity. Convergent validity was acceptable for all factors, except Avoidance. This suggests that the items chosen for the avoidance subscale account for less variance than the total error variance for the factor. However, it has been suggested that if Omega, a measure of composite reliability, is higher than 0.6, in this case 0.79, then the convergent validity of the construct can still be assumed (Fornell and Larcker, 1981). In line with previous research we found that items relating to loss rumination (i.e. dwelling on the circumstances of the death and its outcome) and injustice rumination (i.e. dwelling on how the loss violates rules of fairness) loaded on to separate factors (Eisma et al., 2014).

Cross-lagged analyses indicate that the OG-CS total scale significantly predicts PGD symptoms in both the short term (6–12 months) and long term (12–18 months), after controlling for associations at concurrent and preceding time points. These findings demonstrate that the coping strategies measured in the OG-CS represent a modifiable maintenance factor influencing the trajectory of PGD. Their predictive utility was strongest in the long-term, possibly suggesting that targeted interventions may be most effective after 6–12 months post-death. Moreover, the results highlight a shift in the relationship between grief symptoms and coping strategies over time. Initially, PGD symptoms predict levels of unhelpful coping strategies within the first 6–12 months after loss, suggesting an intertwined and cyclical connection between grief and coping during this period, possibly reflecting a normative grieving process. However, beyond 6–12 months, the influence of PGD symptoms on coping strategies diminishes, indicating a decoupling of the two constructs. This decoupling suggests that while coping strategies initially may be reactive to grief symptoms, they eventually take on a more autonomous role in either perpetuating or mitigating PGD, irrespective of the intensity of grief experienced, perhaps as they become more ingrained overtime. This shift underscores the importance of longitudinal assessment in understanding the transient dynamics of grief and related coping strategies and points to the potential of measuring maladaptive coping as an early indicator of later PGD. This is particularly important given these strategies may offer a clearer path through treatment than a focus solely on symptoms alone. However, further research is needed to explore this.

The pattern of results provide important insights into the temporal development and maintenance of PGD symptoms, lending strong support for the cognitive model for persistent PTSD (Ehlers and Clark, 2000) and the cognitive behavioural model for PGD (Boelen, van den Hout, et al., 2006). Both models underscore the impact of unhelpful coping strategies on symptom onset and maintenance. Moreover, the findings build upon prior research, establishing coping strategies as a causal mediator in the relationship between loss-related memory characteristics, appraisals, and PGD (Smith and Ehlers, 2021a). Due to model complexity the causal mediation analyses were unable to control for autocorrelations and baseline symptoms which may have reduced the predictive utility of the model. The findings reported in this paper confirm the predictive role of coping strategies over time after controlling for baseline symptoms and autocorrelations.

Subscale analyses of the OG-CS and PGD symptoms supported the results of the total scale with the influence of avoidance, proximity seeking and rumination on symptoms of PGD increasing in magnitude over time.

### 4.1 Avoidance

Use of avoidance strategies (0–6 months) early on did not seem to lead to increased PGD symptoms in the short term. However, those with elevated grief early on are more likely to engage in avoidance later. This may point to the oscillation between loss-oriented (loss-focused) and restoration oriented (loss-avoidance) behaviours proposed by the dual process model as being reflective of normative grief adaptation (Stroebe and Schut, 1999). Beyond 6–12 months, however, if individuals are still utilising avoidance strategies to cope with their grief, they are more likely to experience PGD symptoms in the future, suggesting that the dual process model may be limited to the first 6–12 months of bereavement.

Avoidance of reminders of the loss are part of the DSM-5-TR criteria (Prigerson et al., 2021) and are used as a proxy for denial in the ICD-11 criteria (Killikelly et al., 2020). However, its inclusion has proven controversial with studies finding it peripheral to the other PGD symptoms (Stelzer et al., 2020) or finding its exclusion from the criteria does not change their internal consistency or, at best, improves it (Prigerson et al., 2021). The results of the psychometric validation presented in this paper demonstrate a clear and significant relationship between the avoidance strategies measured here and post-loss mental health problems, with longitudinal analyses pointing to their important role in predicting later PGD beyond 6–12 months. This discrepancy may best be explained by considering how avoidance is represented in the different diagnostic conceptualisations of PGD. The DSM-5-TR set of PGD criteria describe avoidance as being driven by a desire to avoid reminders that the person is dead (Prigerson et al., 2021) while measures assessing ICD-11 criteria for PGD ask about reminders of the deceased or the death (e.g. pictures and memories) (Killikelly et al., 2021). It might be that offering a reason for the avoidance (which may differ by individual) or offering limited examples of avoidance (such as memories or photos) may result in reduced endorsement of the symptom and misses the many ways in which people attempt to avoid the reality of the loss. The OG-CS may inform a comprehensive understanding of the avoidant strategies related to loss missed by the assessment of PGD itself.

### 4.2 Proximity seeking

The relationship between proximity seeking and PGD symptoms was significant in both directions at both time points. While the impact of proximity seeking on symptoms was most pronounced in the long term, this difference in strength of association compared to the short term was not statistically significant. These results support previous longitudinal findings from the continuing bonds literature that demonstrated proximity seeking behaviours employed in the months following loss predicted a poorer long-term prognosis (Boelen, Stroebe, et al., 2006; Field et al., 2003; Stroebe et al., 2012). The inverse relationship of symptoms on proximity seeking was significant and stable across time indicating that bereaved people with elevated grief were more likely to engage in proximity seeking to manage their symptoms. This pattern of results fits with a vicious cycle in which maladaptive coping prevents the reduction in symptoms which in turn drives the use of maladaptive coping in order to manage painful symptoms.

### 4.3 Rumination

The results for loss rumination and injustice rumination were similar. Ruminating about the loss was predictive of later PGD symptoms according to the ICD-11 diagnosis in both the short and long term but only in the long-term for DSM-5-TR PGD. As with the other subscales the influence of rumination on symptoms was strongest after 6–12 months had passed. In fact, results demonstrated that while a bidirectional relationship between symptoms and rumination was present early on, rumination was shown to uniquely drive symptoms in the long-term. While many studies have found associations between rumination post-loss and mental health problems, longitudinal investigations demonstrating a prospective influence have been limited (Eisma et al., 2015; Nolen-Hoeksema et al., 1997, 1994; van der Houwen et al., 2010). These findings contradict a recent cross-lagged study that found no effect of depressive rumination on later post-loss psychopathology (Eisma et al., 2022). Eisma and colleagues assessed bereaved adults in their first year of loss and followed them every 6 weeks for just over a year. They found that, similar to our results, symptoms of PGD predicted later use of rumination but found no reciprocal relationship for rumination on symptoms. These findings highlight the importance of the type of repetitive thought in question when investigating rumination in bereavement. Eisma and colleagues used the Ruminative Response Scale of the Response Styles questionnaire (RRS-RSQ, Nolen-Hoeksema and Morrow, 1991) which includes a measure of repetitive thinking not specifically tied to loss (e.g. How often do you think “Why did I deserve this?” or “Why do I always react this way?”) which may not have adequately targeted the specific repetitive thoughts utilized by bereaved people. Eisma et al. (2022) conclude that specific types of grief-related rumination are likely to be more promising treatment targets than depressive rumination. Crucially, the rumination items chosen for the OG-CS were derived from interviews with bereaved people with and without PGD (Smith, 2018) and previous research on rumination (Eisma et al., 2015) and as such, may reflect bereavement-specific repetitive thinking.

Several limitations are worth noting. First, the sample was predominantly White and female, which may limit generalizability. Further research should aim to distribute the measure with more diverse populations to ensure applicability. Second, the study used online self-report measures to assess PGD levels which precludes any conclusions about probable diagnoses. Previous research has highlighted a discrepancy between diagnostic assessments and self-report measures more widely (Krebber et al., 2014) and specifically in the bereavement context (Lenferink et al., 2019). However, diagnostic interviews are time and labour-intensive and as such collecting large samples longitudinally may be difficult. Future research that utilizes the OG-CS within a context where diagnostic interviews are being administered would add weight to the findings presented in this report although we note that currently there are no validated clinical interviews for PGD. Finally, the PGD ICD-11 diagnostic criteria were approximated using an item from a validated PTSD measure, which may have introduced some measurement error. Given validated scales are now available for the ICD-11 criteria (Hyland et al., 2023; Killikelly et al., 2020) further studies should aim to utilize these in the continued investigation of the OG-CS.

The psychometric properties of the OG-CS and its comprehensive measurement of a variety of loss-related coping strategies coupled with its utility in predicting prospective symptoms make it a useful tool for clinicians and researchers in the assessment and treatment of PGD.

## Funding

This work was supported by the 10.13039/100010269Wellcome Trust [AE:200796]; the 10.13039/501100000265Medical Research Council [KS: MR/V001841/1]; 10.13039/100011705MQ [JW: MQ CQRO1260] the 10.13039/501100000272National Institute for Health Research (NIHR) Biomedical Research Centre, based at Oxford University Hospitals NHS Trust [KS: NIHR-INF-0085], and the Oxford Health NIHR Biomedical Research Centre (AE, JW, and KS: NIHR203316) and NIHR Senior Fellowship (AE). The views expressed are those of the author(s) and not necessarily those of the NIHR or the Department of Health and Social Care. These funding sources did not have a role in the design of the study, collection, analysis, and interpretation of the data and in writing the manuscript.

## CRediT authorship contribution statement

**Kirsten V. Smith:** Writing – review & editing, Writing – original draft, Project administration, Methodology, Formal analysis, Data curation, Conceptualization. **Jennifer Wild:** Writing – review & editing, Supervision, Conceptualization. **Anke Ehlers:** Writing – review & editing, Supervision, Methodology, Investigation, Funding acquisition, Conceptualization.

## Declaration of competing interest

The authors declare that they have no known competing financial interests or personal relationships that could have appeared to influence the work reported in this paper.

## Appendix Supplementary materials

Image, application 1
