# Supplementary material for: From loss to disorder: The influence of maladaptive coping on prolonged grief
Source: Psychiatry Res. 2024 Sep;339:116060. doi: 10.1016/j.psychres.2024.116060 (PMC11513616; doi:10.1016/j.psychres.2024.116060)
Supplement: Supplementary file 1 [file mmc1.docx]

Supplementary material

Table A1. Mean scores of variables over time.

| Variable | Time point 1 (0-6 months)  Mean (SD) | | Time point 2 (6-12 months)  Mean (SD) | | Time point 3 (12-18 months)  Mean (SD) | |
| --- | --- | --- | --- | --- | --- | --- |
| PGD ICD-11 | 39.39 | (10.41) | 33.51 | (10.53) | 30.11 | (10.89) |
| PGD DSM-5-TR | 33.31 | (9.36) | 28.32 | (9.48) | 25.37 | (9.70) |
| OG-CS – Total scale | 54.24 | (19.14) | 48.13 | (17.95) | 45.99 | (17.57) |
| OG-CS – Avoidance subscale | 13.24 | (5.18) | 11.95 | (4.75) | 11.34 | (4.79) |
| OG-CS – Proximity seeking subscale | 16.57 | (6.30) | 14.80 | (6.04) | 13.91 | (5.57) |
| OG-CS – Loss rumination subscale | 16.40 | (7.09) | 14.48 | (9.48) | 14.16 | (9.70) |
| OG-CS – Injustice rumination subscale | 7.96 | (3.65) | 6.89 | (3.52) | 6.58 | (3.41) |

Note: PGD ICD-11 and PGD DSM-5-TR measured using the adapted PG-13. OG-CS = The Oxford Grief Coping Strategies Scale.


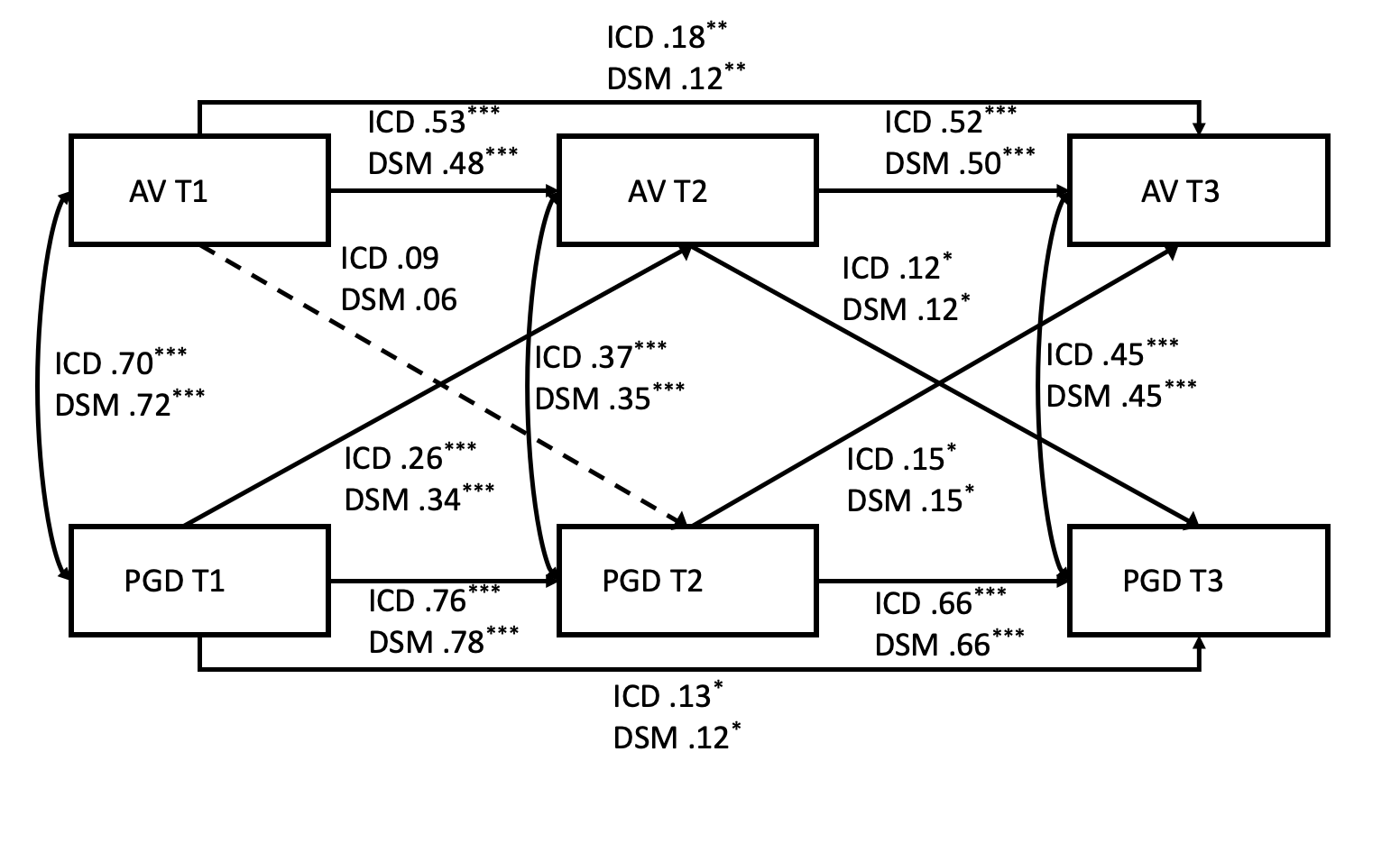


Figure 1A. Avoidance subscale DSM-5-TR, χ^2^ = 0.81, *df* = 2, *p* > 0.05, RMSEA = 0.00 (0.00–0.09), CFI = 1.00, TLI=1.01; ICD-11, χ^2^ = 1.42, *df* = 2, *p* > 0.05, RMSEA = 0.00 (0.00–0.11), CFI = 1.00, TLI=1.00


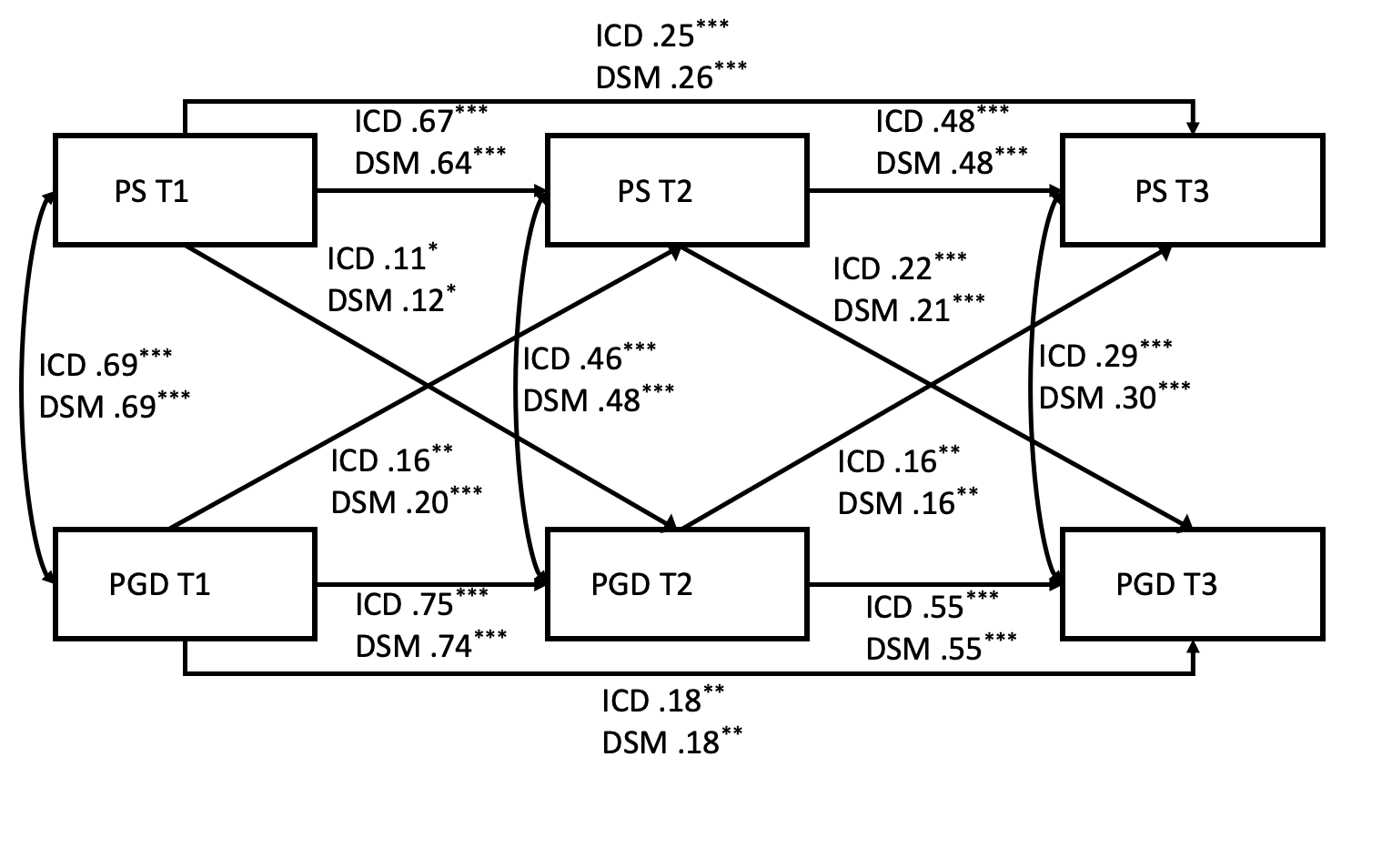


Figure 2A. Proximity seeking subscale DSM-5-TR, χ^2^ = 5.85, *df* = 2, *p* > 0.05, RMSEA = 0.08 (0.00–0.17), CFI = 1.00, TLI=0.98; ICD-11, χ^2^ = 3.91, *df* = 2, *p* > 0.05, RMSEA = 0.06 (0.00–0.11), CFI = 1.00, TLI=.99


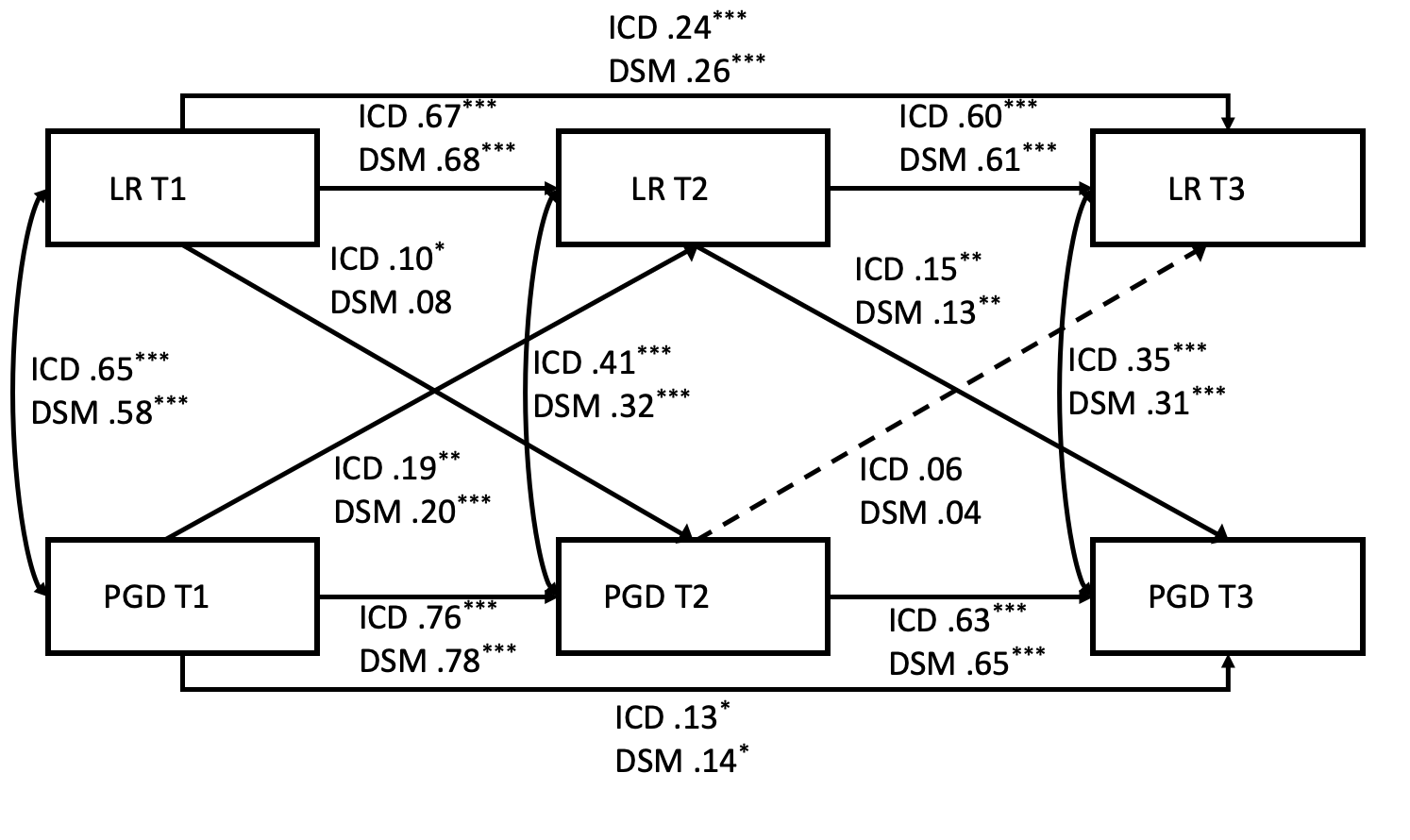


Figure 3A. Loss rumination subscale DSM-5-TR, χ^2^ = 0.13, *df* = 2, *p* > 0.05, RMSEA = 0.00 (0.00–0.03), CFI = 1.00, TLI=1.01; ICD-11, χ^2^ = 0.46, *df* = 2, *p* > 0.05, RMSEA = 0.08 (0.00–0.11), CFI = 1.00, TLI=1.01


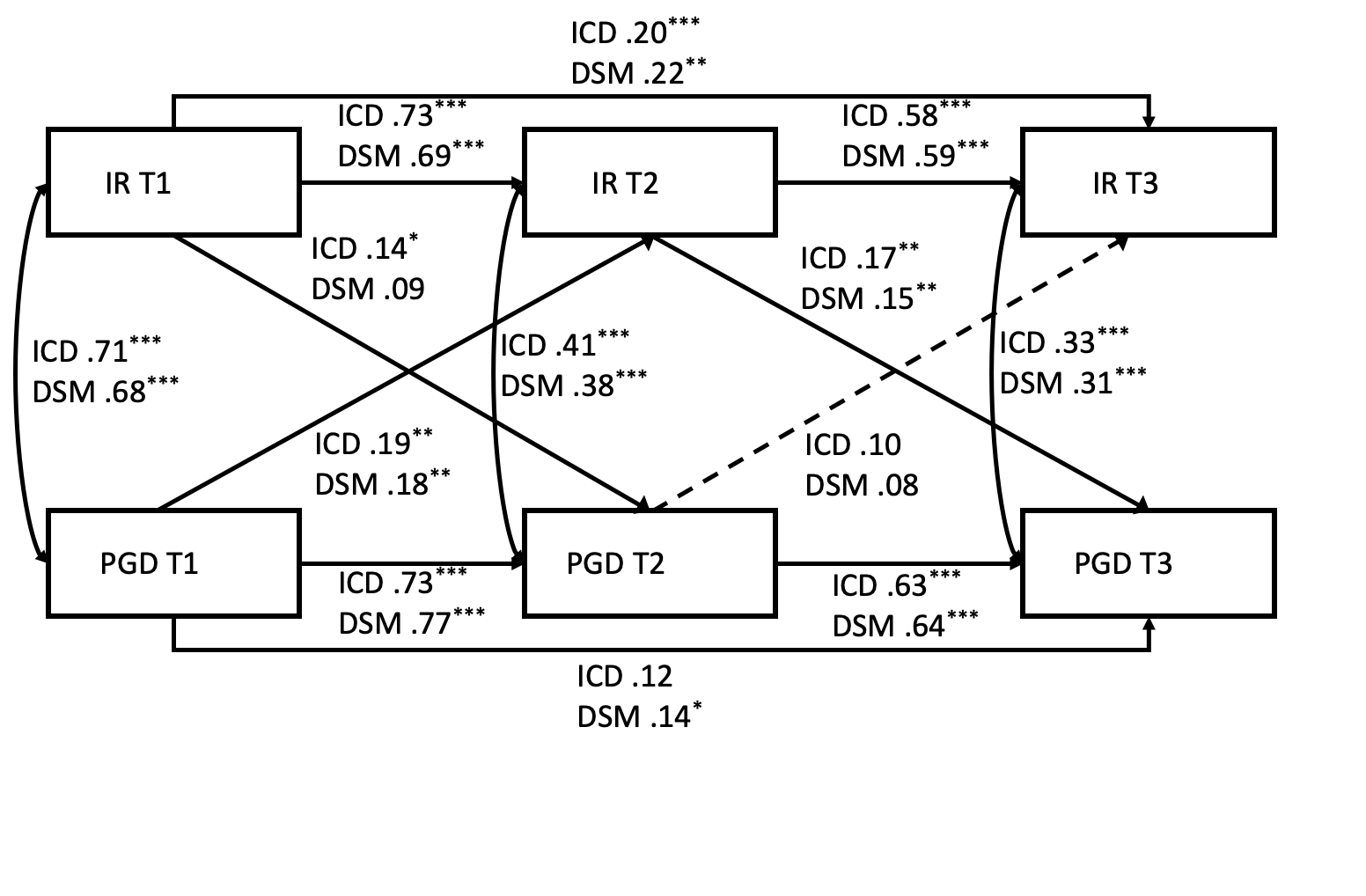


Figure 4A. Injustice rumination subscale DSM-5-TR, χ^2^ = 1.34, *df* = 2, *p* > 0.05, RMSEA = 0.00 (0.00–0.11), CFI = 1.00, TLI=1.01; ICD-11, χ^2^ = 2.37, *df* = 2, *p* > 0.05, RMSEA = 0.03 (0.00–0.13), CFI = 1.00, TLI=1.00

**The Oxford Coping Strategies Scale (OG-CS)**

Kirsten V. Smith, DClinPsy. DPhil., Jennifer Wild, DClinPsy., Anke Ehlers, PhD.

People try to cope with loss in different ways. Below you will find a range of statements describing different ways of coping. Please indicate below to what extent each statement applies to you in the PAST MONTH. **There are no right or wrong** **answers**, we are interested in your personal reaction.

|  | **Not at all**  **(0)** | **Sometimes**  **(1)** | **About half the time**  **(2)** | **Most of the time**  **(3)** | **Always**  **(4)** |
| --- | --- | --- | --- | --- | --- |
| 1. I avoid watching television programmes that remind me of [-] or death in general. |  |  |  |  |  |
| 2. I avoid places we went together. |  |  |  |  |  |
| 3. I avoid eating foods and meals that we shared or [-] liked. |  |  |  |  |  |
| 4. I avoid making any changes to my life since [-] ’s death. |  |  |  |  |  |
| 5. I make an effort to hold back my feelings. |  |  |  |  |  |
| 6. In the company of others I try hard to stop myself from breaking down. |  |  |  |  |  |
| 7. I feel compelled to surround myself with things that they liked. |  |  |  |  |  |
| 8. I bring images of [-] to mind. |  |  |  |  |  |
| 9. I am still carrying out a routine as a way of caring for them. |  |  |  |  |  |
| 10. I neglect other things because I spend a lot of time doing things for [-] (e.g. creating memorials, fundraising). |  |  |  |  |  |
| 11. I feel compelled to touch things that [-] touched (e.g. belongings, chairs, beds). |  |  |  |  |  |
| 12. I spend a lot of time thinking about joining [-] (in the afterlife). |  |  |  |  |  |
| 13. I dwell on the things we won't get to do together. |  |  |  |  |  |
| 14. I can’t stop thinking about how afraid [-] was. |  |  |  |  |  |
| 15. I think over and over about how others failed to ease [-]'s suffering.^a^ |  |  |  |  |  |
| 16. I think over and over about what I could have done to prevent [-]'s death/ease their suffering.^a^ |  |  |  |  |  |
| 17. I go over and over how our last moments could have been more fulfilling. |  |  |  |  |  |
| 18. I dwell on moments that could have changed the outcome. |  |  |  |  |  |
| 19. I can’t stop thinking about how much [-] suffered. |  |  |  |  |  |
| 20. I worry that [-] has not found peace (in the afterlife). |  |  |  |  |  |
| 21. I ask myself why I deserved this loss.^a^ |  |  |  |  |  |
| 22. I think about the unfairness of the loss.^a^ |  |  |  |  |  |
| 23. I think over and over about how it could be that this happened. |  |  |  |  |  |

Please note anything else that you feel is important below.

____________________________________________________________

________________________________________________________________

Avoidance subscale: 1,2,3,4,5,6

Proximity seeking subscale: 7,8,9,10,11,12,13

Loss rumination: 14,15,16,17,18,19,20

Injustice rumination: 21,22,23

Scoring note for items 12 and 20: Religiosity can be taken into account (i.e. a non-religious person would not see the words “in the afterlife” at the end of the item).
